# The Stimulus to Growth

**Published:** 1920-08-07

**Authors:** 


### EXPERIMENTAL STUDIES IN CANCER.

### II.—The Stimulus to Growth.

Positive results have been obtained in the production of mouse adenosarcomata by means of trauma and pressure, but as a rule such attempts have caused no more than a proliferation of the epithelium, which, however, when the irritant, such as paraffin or soot, is removed has returned to its normal condition. Nevertheless, why all pipe-smokers do not develop carcinoma of the tongue is still as unexplained as the fact that domestic animals, *e.g.*, horses, do not more frequently develop irritant malignant overgrowths. One striking example there is in the effects of *x*-ray as shown by the unfortunate and unintentional experiments on man by the early workers in radiology, and it is of great interest that sarcoma has been deliberately produced in the rat by the same means. Recently Japanese investigations have caused the development of carcinoma by repeatedly painting rabbits' ears with tar, and it is particularly noteworthy that some of these growths actually produced metastases. In the same irritational class can be placed, too, the gastric rate carcinomas studied by Febiger, and associated with the remains of a parasitic worm. This worm has a certain species of cockroach as its intermediate host and by feeding rats on infected insects, Febiger was able to see carcinomata develop in many of his animals. More recently similar growths in rats have been seen to develop around the encysted stage of a particular tape worm, and in view of the previously isolated transplantable growths, it is of particular interest that a number of the more recently discovered types are readily passed on in the same way.

### Heredity and Contagion.

The general conclusion upon family liability to cancer, a subject which is always wrapt in considerable uncertainty, are set out as follows: "Study of tumour statistics in man has not yielded positive conclusions as to the contagiousness of cancer, the existence of so-called cancer-houses or communities, or as to the hereditary tendency of malignant disease. The study of tumours in animals on the large scale, and especially with animals which pass through many generations during the lifetime of a single observer, has shown an absence of anything like a contagious nature of neoplasms and an absence of anything corresponding to a cancerous house or community."

Another point which animal experiment has demonstrated came with the proof that massage and manipulation of all these growths is a potent factor in producing secondary deposits elsewhere in the system, but that partial surgical removal and disturbance does not necessarily increase this tendency. The number and size of metastases depend apparently upon the time a tumour has been growing and upon its actual size, as well as its particular cell-type, so that we come to view malignancy more and more as a mechanical and structural property of the growth as a whole rather than a particular function possessed by the "malignant cell." But that the cancer cell has certain specific habits and possibly secretions seems to be almost an established fact.

### The Properties of the Cancer Cell.

What we may call "cellular immunity" is quite definite. It is nearly always impossible to transplant a mouse cancer into a rat, and *vice versâ*. There is a definite immunity of species. Even the few attempts which were made to transfer human tumours to anthropoid apes have been utterly unsuccessful. Also a specific cellular effect is noted in the extremely common lymphocytosis which is seen generally and locally in a great majority of cancerous animals. It has, in fact, been possible to give mice a high degree of artificial immunity by first producing a lymphocytosis by subjecting them to a temperature of 55 to 63 degrees for five minutes and then transplanting a piece of tumour. This lymphocytic immunity is curiously anomalous in view of the common occurrence of metastases in the lymph glands themselves!

In any case, experimental tumour immunity seems to be entirely cellulous. No immune bodies in the sera have ever been found. Serum from immune animals has no protective effect when injected into others. In vitro tissue cultures of cancer cells can be as successfully made in the plasma of immune animals as in the plasma of susceptible animals. Nevertheless, if a transplanted tumour does not develop in a susceptible animal, but instead retrogresses and dies, just as in many similar sub-lethal bacteriological inoculations, a high degree of immunity is developed. Exactly where the immune substance resides is at present something of a mystery, but at least it appears to have a cellular and not a serum origin.

Another interesting proof has come from animal work. Tumour grafts previously subjected to *x*-rays or radium are extremely loth to grow. If the tumour graft is inoculated and then rayed, a retarding effect is noted, but it is now much less marked. Apparently, although the cancerous tissue is more susceptible than normal tissue to the rays, yet the difference is not sufficient to make the action selective, except in the case of very superficial tumours. And so one might proceed with a number of illustrative descriptions of the enormous number of experiments which are bringing us daily nearer to a proper understanding of, not so much what is the "Cause of Cancer," but what are the factors which can provide that stimulus to the parent cell necessary to provoke tumour formation. At present experiment is telling us more and more of the way in which growth proceeds, and the factor which influences it. Gradually it is working back to an understanding of the possible factors which may form an original cause. Probably there is no single cause—in the sense of a "causal organism."

The first article appeared on July 31, p. 451.

